# Supplementary material for: Physically-interpretable classification of biological network dynamics for complex collective motions
Source: Sci Rep. 2020 Feb 20;10:3005. doi: 10.1038/s41598-020-58064-w (PMC7033192; doi:10.1038/s41598-020-58064-w)
Supplement: Supplementary file 1 — Supplementary Information. [file 41598_2020_58064_MOESM1_ESM.pdf]

Supplementary materials for:

Physically-interpretable classification of biological network dynamics for complex collective motions

Keisuke Fujii, Naoya Takeishi, Motokazu Hojo, Yuki Inaba, Yoshinobu Kawahara

Text 1. Details of classification.

Classification using Graph DMD modes

After Graph DMD, we perform classification in three different approaches. The first two approaches create feature vectors and the last is a neural network approach to automatically create the features. The first is simply to vectorise the Graph DMD modes (denoted as GDMD spectrum) to directly reflect the node (i.e. players) information. We used the elements of the modes regarding the relation among defenders, among attackers and defenders and among defenders and ring (as geometric information) as a feature vector for the team-defence recognition task, and those among attackers and among attackers and defenders (without geometric information) for the team-offence recognition task. The last two is to compute graph features by existing methods: the second is graph Laplacian eigenvalues[1] as a well-known baseline feature of a graph[2] (denoted as GDMD Laplacian) and the third is deep graph convolutional neural networks[3] (denoted as GDMD GCN) as a recent promising method to classify the graph data.

For a brief explanation of Graph Laplacian, let $\boldsymbol{A} \in \mathbb{R}^{m \times m}$ be an adjacency matrix in an undirected graph, which has elements $A_{i,j}$ for $i, j = 1, \ldots, m$. Using a degree matrix $\boldsymbol{D}$, which is a diagonal matrix having elements $D_{i,i} = \sum_{j=1}^{m} A_{i,j}$, Graph Laplacian matrix is defined as $\boldsymbol{L} = \boldsymbol{D} - \boldsymbol{A}$. We computed normalised Graph Laplacian defined as $\mathscr{L} = \boldsymbol{D}^{-1/2}\boldsymbol{L}\boldsymbol{D}^{-1/2}$. We used the averaged Graph DMD modes as the adjacency matrix $\boldsymbol{A}$. We then obtained k positive smallest eigenvalues of the normalised graph Laplacians[2]. We set $k = 10$, which means all eigenvalues except 0 regarding the averaged Graph DMD modes (the number of the nodes $m = 11$). Then, we used the eigenvalues in ascending order as an input of the classifier. This approach extracts the graph topology without the node information.

GCN is a generalised convolutional neural network framework to graphs in the spectral domain. The GCN we used[3] keeps more node information and learn the global node topology by sorting a graph's node in a consistent order called SortPooling layer, so that traditional neural networks can be trained on the graphs. This GCN is closely related to some type of graph kernels based on structure propagation, especially the Weisfeiler-Lehman subtree kernel[4] and propagation kernel[5]. The previous work[3] showed that the GCN achieved highly competitive classification performance with various graph kernels and neural network methods. Thus, we used this method as a recent promising method to classify the graph data. We used the averaged Graph DMD modes as an input adjacency matrix and default parameters in open source code `https://github.com/muhanzhang/DGCNN`.

For all classification methods except for the neural network approaches, logistic regression was adopted as a linear binary classification model. For the neural network approaches[3,6], we used the default softmax layer as classifiers.

Classification using other methods

As comparable methods to classify graph sequence data, we adopted four methods. The first is a method using vectorised basic DMD[7] modes (denoted as DMD spectrum) as a baseline of DMD approaches (the selection of the elements was the same as GDMD spectrum). The second is the Koopman spectral kernel[8] using DMD with reproducing kernels[9] as the existing method[10] to classify the collective motion dynamics (denoted as KDMD spectral kernel). In the two methods, input data should be matrix thus we reshaped the input adjacency matrix series to the matrix in which rows and columns were temporal stamps and vectorised adjacency matrix, respectively. Koopman spectral kernels generalised a kernel[11] between dynamical systems to nonlinear dynamical systems. Among the above kernels, we used Koopman kernel of principal angle between the subspaces of the estimated Koopman mode, showing the best discriminative performance[8] using the Koopman modes given by DMD with reproducing kernels. Regarding DMD with reproducing kernels[9], we adopted the Gaussian kernel and the kernel width was set as the median of the distances from data.

The third is a hand-crafted feature as a simple baseline method, consisted of vectorised temporal

average, maximum and minimum values of the elements of input adjacency matrix series. Fourth is an advanced neural network approach to classify graph sequence data called spatio-temporal GCN[6] as a recent promising end-to-end method. Spatio-temporal GCN is a graph-based neural network for action recognition by modelling dynamic skeletons with the joints as graph nodes and natural connectivities in both human body structures and time as graph edges. That is, the input of the original work is the joint coordinate vectors on the graph nodes. Multiple layers of spatio-temporal graph convolution operations will generate higher-level feature maps on the graph. We used the sequence of adjacency matrices as the input and basically used default parameters in opensource code `https://github.com/yysijie/st-gcn`, except for the below parameters. To adjust the model to the collective motion in this study, since indices of players were based on their distance from the ball and teammates, the neighbour edges were defined as the nearest attackers and defenders, and the ring and players. Moreover, for higher-level spatio-temporal graph convolution, we adopted spatial configuration partitioning, which shows the best action recognition performance among various partitioning strategy[6]. The strategy divides the neighbour set into three subsets: the root node itself, centripetal group and centrifugal group. This enables us to semantically higher-level spatio-temporal graph convolution.

Text 2. Selection of Graph DMD parameters.

Here, we describe the selection of parameters regarding Graph DMD (the Graph DMD tolerance, the temporal window size and the cutoff frequency) and quantitatively validated the applicability of Graph DMD to the sport data in terms of reconstruction error using a validation dataset.

We used the absolute reconstruction error defined as $(1/m^2\tau)\sum_{t=0}^{\tau-1}\sum_{i=1}^{m}\sum_{j=1}^{m}\|A_{i,j,t}-\hat{A}_{i,j,t}\|$, where $\hat{A}_{i,j,t}$ is a reconstructed element corresponding to $A_{i,j,t}$. In Graph DMD, Graph DMD tolerance ε (i.e. the tolerance in the successive SVD in tensor-train decomposition) is critical for the data reconstruction. We set $\varepsilon = 1.0 \times 10^{-5}$ because Supplementary Fig. 1a shows that the reconstruction error in this tolerance is lower than other values.

Next, we performed Graph DMD in sliding temporal windows, because complex collective motions often transiently change their rules of motions. For basic DMD, researchers used sliding windows applied to cortical electroencephalogram data[12]. However, there is a trade-off between the reconstruction error and the meaningful dynamical information in the time interval. If the window size is too small, the reconstruction error may be small but the extracted information may be useless. If too large, the extracted information may reflect meaningful information but the reconstruction error may be too large. Supplementary Fig. 1b shows that when the window size is 60 frames, the reconstruction error greatly increased. Thus, we set the window size to 50 frames (2 s) including overlaps of 25 frames (1 s).

In addition, a cutoff frequency is also an important parameter. In this study, meaningful motion frequency is considered to be in a low-frequency band (e.g. under 2 Hz) rather than a high-frequency band (e.g. over 2 Hz), when considering the distinction among team-defence or offence motions. Thus, obtained data was first low-pass filtered at the cutoff frequency. Moreover, since DMDs are not guaranteed to extract the dynamics within the frequency band, we averaged Graph DMD modes within the temporal frequency band using DMD eigenvalues. The selection of the cutoff frequency also has a trade-off between the reconstruction error and meaningful dynamical information. Supplementary Fig. 1c shows that when the cutoff frequency was 3 Hz, the reconstruction error greatly increased. Thus, we set the cutoff frequency to 2 Hz.

Text 3. Additional analysis of the number of Graph DMD modes and classification performances.

After reviewers' comment, we additionally analyzed the effects of Graph DMD parameters on (i) the number of Graph DMD modes and (ii) classification performances.

First, regarding the number of Graph DMD modes, the maximal number of DMD modes is algorithmically limited to $min(m^2, \tau - 1)$ where m is the number of agents and the reference point and τ is length of the sequences in a sliding window. The tolerance eliminated the number of DMD modes with small contributions in the Graph DMD algorithm (more accurately, in the tensor-train decomposition). Thereafter, we removed the DMD modes over 2 Hz (referred to as the cutoff frequency) based on the result in Supplementary Fig. 1c. Thus, we analyzed the number of remaining DMD modes after these eliminations.

We investigated the effects of Graph DMD parameters on the number of Graph DMD modes using the validation data and the same sets of parameters in Supplementary Fig. 1. Results show that monotonic decrease or increase in the number of DMD modes for various Graph DMD tolerances (Supplementary Fig. 3a), sliding window sizes (Supplementary Fig. 3b) and cutoff frequencies (Supplementary Fig. 3c). These results follow our intuition, because the increases of window size and cutoff frequency directly increase the number of Graph DMD modes, and the increase of the tolerance eliminates the modes with small contributions. Additionally, we investigated the effect of the ring information on the number of Graph DMD modes. The results in Supplementary Fig. 4 were similar to those in Supplementary Fig. 3.

Second, we investigated the effect of $\sigma = \sigma'^2/2\log 2$ (i.e. a variable in the denominator in the exponential in Eq. (5)) and the ring information on the classification performances using the test dataset. As shown in Supplementary Table 3, the classification performances when $\sigma' = 1, 2$ and those without the ring information were worse than that when $\sigma' = 1.5$ and those with ring information. Compared with Table 1, these differences in σ' were smaller than the differences between our method and other methods. Additionally, the performances without the ring information were worse than other methods such as Graph DMD Laplacian and hand-crafted feature. These results suggest that appropriate σ and the ring information contributed to the classification performance.

References

1. Chung, F. R. *Spectral graph theory*. 92 (American Mathematical Soc., 1997).
2. de Lara, N. & Pineau, E. A simple baseline algorithm for graph classification. *arXiv preprint arXiv:1810.09155* (2018).
3. Zhang, M., Cui, Z., Neumann, M. & Chen, Y. An end-to-end deep learning architecture for graph classification. In *Thirty-Second AAAI Conference on Artificial Intelligence*, 4438–4445 (2018).
4. Shervashidze, N., Schweitzer, P., Leeuwen, E. J. v., Mehlhorn, K. & Borgwardt, K. M. Weisfeiler-lehman graph kernels. *J. Mach. Learn. Res.* **12**, 2539–2561 (2011).
5. Neumann, M., Garnett, R., Bauckhage, C. & Kersting, K. Propagation kernels: efficient graph kernels from propagated information. *Mach. Learn.* **102**, 209–245 (2016).
6. Yan, S., Xiong, Y. & Lin, D. Spatial temporal graph convolutional networks for skeleton-based action recognition. In *Thirty-Second AAAI Conference on Artificial Intelligence*, 7444–7452 (2018).
7. Tu, J. H., Rowley, C. W., Luchtenburg, D. M., Brunton, S. L. & Kutz, J. N. On dynamic mode decomposition: Theory and applications. *J. Comput. Dyn.* **1**, 391–421 (2014).
8. Fujii, K., Inaba, Y. & Kawahara, Y. Koopman spectral kernels for comparing complex dynamics: Application to multiagent sport plays. In *European Conference on Machine Learning and Knowledge Discovery in Databases (ECML-PKDD'17)*, 127–139 (Springer, 2017).
9. Kawahara, Y. Dynamic mode decomposition with reproducing kernels for koopman spectral analysis. In *Advances in Neural Information Processing Systems 29*, 911–919 (2016).
10. Fujii, K., Kawasaki, T., Inaba, Y. & Kawahara, Y. Prediction and classification in equation-free collective motion dynamics. *PLoS Comput. Biol.* **14**, e1006545 (2018).
11. Vishwanathan, S., Smola, A. J. & Vidal, R. Binet-cauchy kernels on dynamical systems and its application to the analysis of dynamic scenes. *Int. J. Comput. Vis.* **73**, 95–119 (2007).
12. Brunton, B. W., Johnson, L. A., Ojemann, J. G. & Kutz, J. N. Extracting spatial-temporal coherent patterns in large-scale neural recordings using dynamic mode decomposition. *J. Neurosci. Methods* **258**, 1–15 (2016).

Supplementary Figures

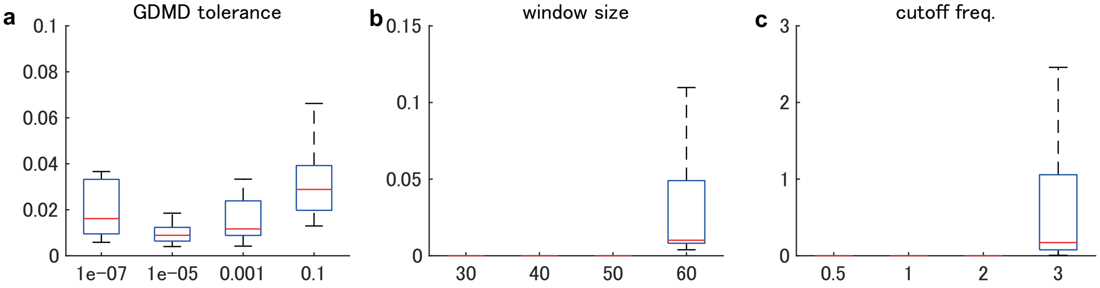

Figure S1. Reconstruction errors of Graph DMD among various parameters. Reconstruction errors of Graph DMD among various parameters: (a) Graph DMD tolerance, (b) sliding window size and (c) cut-off frequency of low-pass filter.

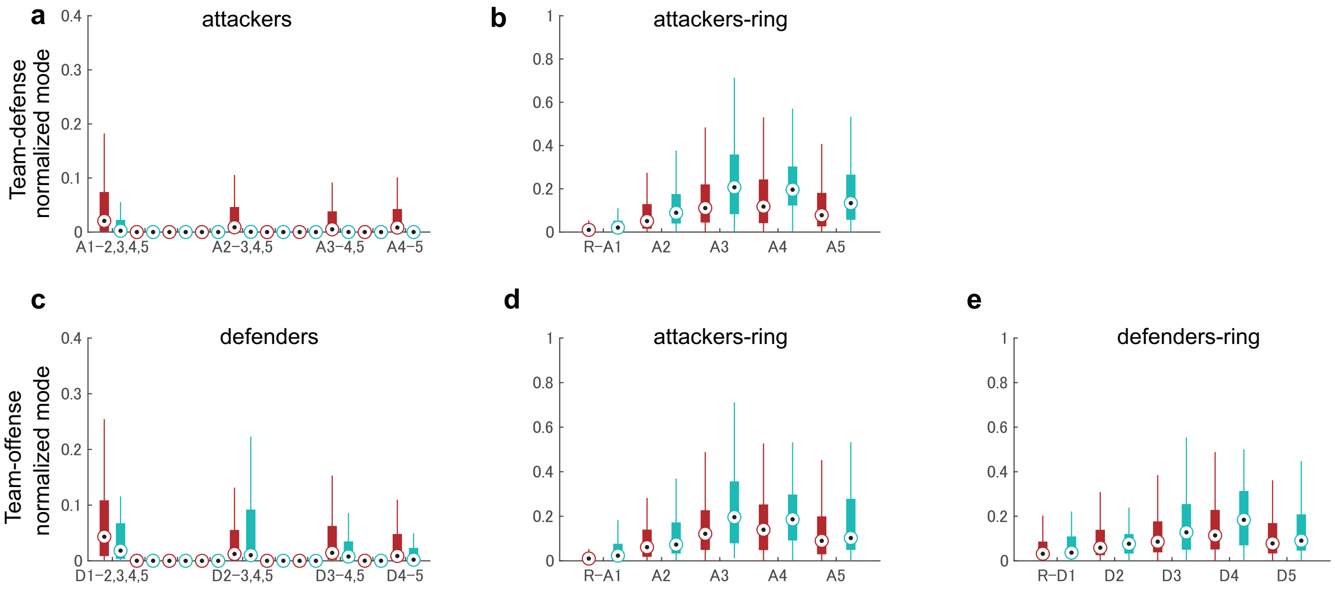

Figure S2. Boxplots for the elements of Graph DMD modes. Boxplots for the elements of Graph DMD modes for label 1 (red) and 2 (blue) which were not used as feature vectors in team-defense (a-c) and offense (d and e) recognition tasks are shown. These plots are the elements of GDMD modes among attackers (a), and between attackers and the ring (b) in team-defense recognition task, and those among defenders (c), between attackers and the ring (d) and between defenders and the ring (e) in team-offense recognition task. Notations are same as Fig. 5.

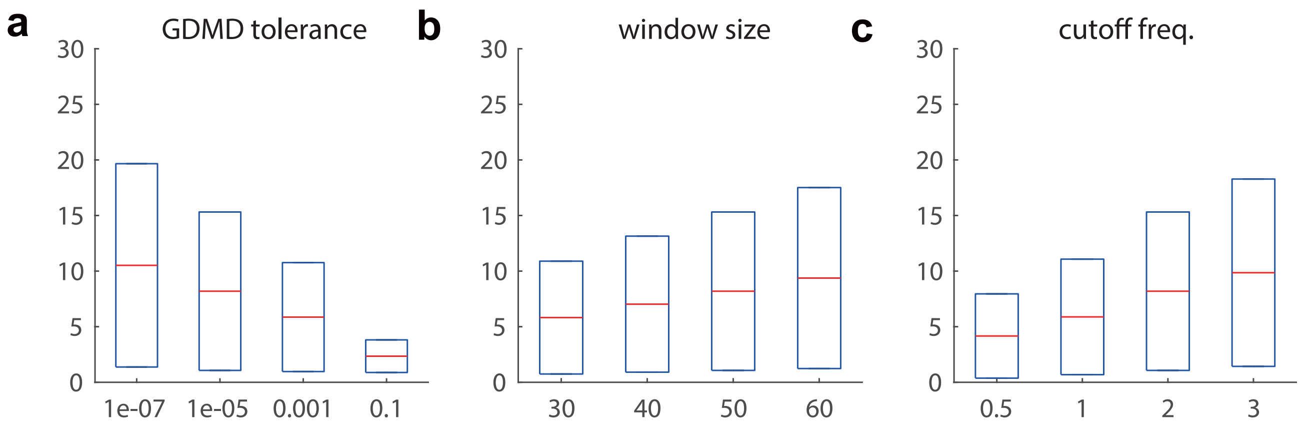

Figure S3. Numbers of Graph DMD modes among various parameters. Numbers of Graph DMD among various parameters: (a) Graph DMD tolerance, (b) sliding window size and (c) cut-off frequency of low-pass filter.

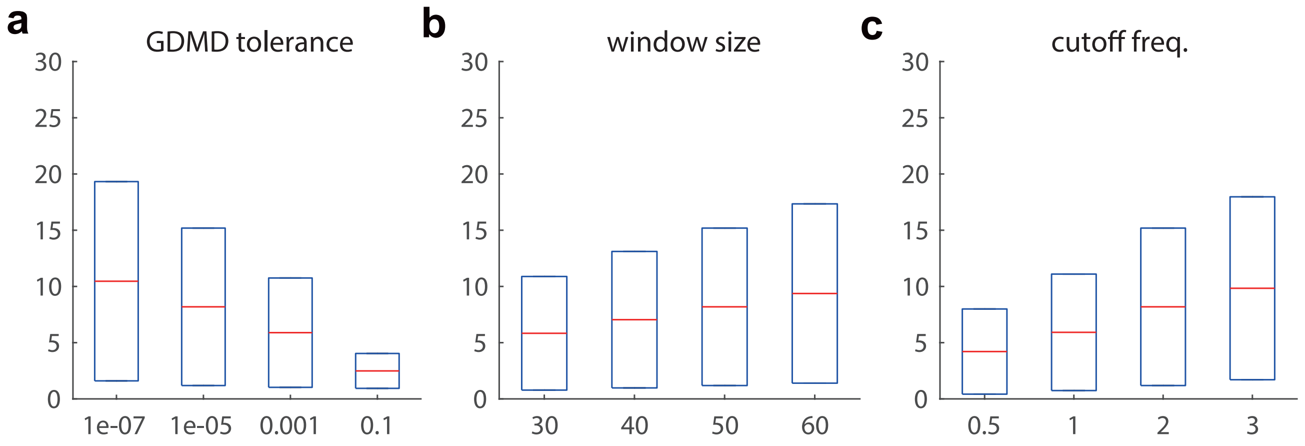

Figure S4. Numbers of Graph DMD among various parameters. Numbers of Graph DMD among various parameters: (a) Graph DMD tolerance, (b) sliding window size and (c) cut-off frequency of low-pass filter.

Supplementary Tables

	Odds ratio	p	Confidence interval	
D1-D2	31.815	0.022	0.492	6.428
D1-D3	4.59×10^9	0.040	0.990	43.508
D1-D4	< 0.001	0.423	−48.727	20.458
D1-D5	< 0.001	0.626	−83.331	50.154
D2-D3	0.859	0.927	−3.403	3.100
D2-D4	0.038	0.494	−12.664	6.113
D2-D5	< 0.001	0.112	−59.429	6.184
D3-D4	6.252	0.367	−2.153	5.819
D3-D5	0.157	0.633	−9.470	5.763
D4-D5	8538.934	< 0.001	4.774	13.331
A1-D1	1.485	0.737	−1.908	2.699
A1-D2	0.568	0.637	−2.915	1.784
A1-D3	0.492	0.532	−2.934	1.516
A1-D4	0.029	0.011	−6.245	−0.805
A1-D5	21.537	0.009	0.752	5.387
A2-D1	5.692	0.175	−0.773	4.251
A2-D2	0.785	0.812	−2.239	1.755
A2-D3	1.844	0.563	−1.462	2.686
A2-D4	0.637	0.686	−2.633	1.732
A2-D5	15.120	0.024	0.362	5.070
A3-D1	0.102	0.045	−4.503	−0.053
A3-D2	0.445	0.505	−3.195	1.574
A3-D3	6.438	0.097	−0.337	4.062
A3-D4	0.028	0.001	−5.719	−1.408
A3-D5	1.097	0.941	−2.362	2.546
A4-D1	22.217	0.017	0.562	5.640
A4-D2	0.993	0.995	−2.185	2.170
A4-D3	4.812	0.187	−0.764	3.907
A4-D4	3.795	0.254	−0.959	3.626
A4-D5	2.206	0.511	−1.566	3.148
A5-D1	0.671	0.736	−2.720	1.922
A5-D2	19.102	0.068	−0.213	6.113
A5-D3	1.449	0.825	−2.911	3.652
A5-D4	0.281	0.398	−4.218	1.676
A5-D5	195.490	< 0.001	2.783	7.768
Ring-D1	0.002	< 0.001	−9.220	−2.828
Ring-D2	0.030	0.049	−6.966	−0.023
Ring-D3	0.136	0.188	−4.965	0.977
Ring-D4	28.668	0.017	0.593	6.118
Ring-D5	0.126	0.153	−4.912	0.770

Table S1. **Results of logistic regression in team-defense recognition task.**

	Odds ratio	p	Confidence interval	
A1-A2	1.06×10^4	< 0.001	4.770	13.761
A1-A3	3.32×10^{30}	0.249	−49.088	189.643
A1-A4	7.26×10^{18}	0.635	−136.033	222.890
A1-A5	23.986	0.979	−238.609	244.964
A2-A3	11.972	0.266	−1.894	6.859
A2-A4	5.631	0.792	−11.148	14.605
A2-A5	< 0.001	0.219	−481.691	110.421
A3-A4	44.656	0.135	−1.177	8.775
A3-A5	0.292	0.840	−13.176	10.714
A4-A5	18.181	0.135	−0.899	6.700
A1-D1	0.139	0.111	−4.396	0.452
A1-D2	0.163	0.128	−4.152	0.522
A1-D3	2.588	0.391	−1.224	3.125
A1-D4	0.080	0.055	−5.102	0.051
A1-D5	23.595	0.008	0.826	5.496
A2-D1	3.565	0.326	−1.268	3.810
A2-D2	0.909	0.923	−2.009	1.819
A2-D3	2.238	0.459	−1.328	2.940
A2-D4	0.668	0.709	−2.522	1.715
A2-D5	1.749	0.617	−1.630	2.748
A3-D1	0.166	0.102	−3.945	0.359
A3-D2	1.341	0.781	−1.777	2.364
A3-D3	3.155	0.287	−0.966	3.264
A3-D4	0.439	0.447	−2.941	1.296
A3-D5	3.807	0.244	−0.914	3.587
A4-D1	5.382	0.143	−0.571	3.937
A4-D2	2.960	0.334	−1.117	3.288
A4-D3	30.457	0.004	1.112	5.720
A4-D4	2.691	0.397	−1.301	3.281
A4-D5	0.994	0.996	−2.180	2.169
A5-D1	0.425	0.497	−3.327	1.616
A5-D2	2.208	0.603	−2.194	3.778
A5-D3	0.036	0.042	−6.531	−0.116
A5-D4	0.695	0.792	−3.063	2.336
A5-D5	26.988	0.005	0.987	5.604

Table S2. Results of logistic regression in team-offense recognition task.

	Team-defence			Team-offence		
	Acc	AUC	F-measure	Acc	AUC	F-measure
with ring, $\sigma' = 1.5$	0.785 ± 0.062	0.833 ± 0.057	0.533 ± 0.058	0.809 ± 0.046	0.803 ± 0.061	0.455 ± 0.060
with ring, $\sigma' = 1.0$	0.777 ± 0.052	0.801 ± 0.079	0.516 ± 0.061	0.799 ± 0.043	0.773 ± 0.059	0.442 ± 0.049
with ring, $\sigma' = 2.0$	0.784 ± 0.051	0.795 ± 0.063	0.516 ± 0.040	0.787 ± 0.056	0.759 ± 0.070	0.434 ± 0.057
without ring, $\sigma' = 1.5$	0.725 ± 0.052	0.741 ± 0.073	0.473 ± 0.076	0.784 ± 0.050	0.755 ± 0.078	0.424 ± 0.066

Table S3. Classification performance of our method in various parameters after reviewers' comments. Accuracy (Acc), area under the curve (AUC) based on receiver operating characteristic (ROC) curve and F-measure are indicated.
